# The role of radiology in diagnosis of Zinner syndrome in a young man with scrotal pain

**DOI:** 10.1016/j.radcr.2023.08.072

**Published:** 2023-09-07

**Authors:** Ali Zare, Behzad Narouie, Farzad Moloudi, Fatemeh Moosavian, Mohadese Ahmadzade, Hamidreza Rouientan

**Affiliations:** a Department of Urology, Shahid Sadoughi University of Medical Sciences, Yazd, Iran; b Department of Urology, Zahedan University of Medical Sciences, Zahedan, Iran; c Department of Radiology, Urmia University of Medical Sciences, Urmia, Iran; d Department of Radiology, Shahid Beheshti University of Medical Sciences, Tehran, Iran; e Department of Urology, Urology and Nephrology Research Center, Shahid Labbafinejad Medical Center, Shahid Beheshti University of Medical Sciences, Tehran, Iran; f Pardis Noor Medical Imaging Center, Tehran, Iran

**Keywords:** Zinner syndrome, Congenital anomaly, Radiological evaluation

## Abstract

Zinner Syndrome is a rare congenital anomaly. It is considered a rare cause of male infertility and can cause a range of clinical manifestations that may lead to significant morbidity. The diagnosis of Zinner Syndrome requires a high index of suspicion, combined with a detailed clinical evaluation and imaging studies. Ultrasonography, computed tomography, and MRI are the imaging modalities of choice for the diagnosis of this condition. Radiological evaluation also plays a crucial role in the management of Zinner Syndrome. In symptomatic cases, surgical intervention may be necessary, and radiology is essential for surgical planning and postoperative monitoring. In this case report, we describe an uncommon case of a 35-year-old patient with vague scrotal pain and discuss the clinical presentation, diagnosis, and management of this rare condition. Prompt and accurate diagnosis is important to prevent the potential morbidity associated with this condition, such as recurrent epididymitis, urinary tract infections, and infertility.

## Introduction

Zinner Syndrome is a rare congenital anomaly that is characterized by the triad of seminal vesicle cysts, ipsilateral renal agenesis, and ejaculatory duct obstruction. This syndrome was first described by Zinner in 1914 and is considered a rare cause of male infertility. The condition occurs due to a developmental abnormality during the embryonic period, leading to a failure in the normal fusion of the mesonephric ducts, ureteric bud, and Wolffian duct [1].

Although Zinner Syndrome is a very rare condition, it can cause a range of clinical manifestations that may lead to significant morbidity. In many cases, the condition is asymptomatic and is diagnosed incidentally during imaging studies performed for other indications. However, it can cause a variety of clinical manifestations, including recurrent epididymitis, urinary tract infections, and infertility [[1], [2]–3].

The diagnosis of Zinner Syndrome requires a high index of suspicion, combined with a detailed clinical evaluation and imaging studies. Ultrasonography computed tomography (CT) scan, and magnetic resonance imaging (MRI) are the imaging modalities of choice for the diagnosis of this condition. Ultrasonography can identify the absence of the ipsilateral kidney and seminal vesicle cysts, while CT scan and MRI can provide more detailed information about the anatomy of the genitourinary tract and the extent of the cystic lesions [4].

Treatment options for Zinner Syndrome depend on the clinical manifestations and can range from conservative management to surgical intervention. Conservative management may include the use of antibiotics for recurrent urinary tract infections or epididymitis, while surgical intervention may be required for symptomatic seminal vesicle cysts or ejaculatory duct obstruction [5].

In this case report, we describe an uncommon case of a 35-year-old patient with vague scrotal pain and discuss the clinical presentation, diagnosis, and management of this rare condition.

## Case presentation

A 35-year-old man presented to our outpatient urology clinic with a complaint of dull pain in the left testicle, which started about 20 days ago. The pain spread to the inguinal area on the same side. He denied any history of recent trauma to the testicles or abdomen. He also denied any urinary symptoms such as dysuria. The patient was married and infertile, but he had not followed up on the cause of infertility. He denied any history of sexually transmitted infections. His medical history was unremarkable, and he was not taking any medications. On physical examination, his vital signs were stable. Inspection of the genitalia revealed no abnormalities. The testicles had a normal consistency and size, and the cremasteric reflex was present bilaterally. Bilateral vas deferens were palpated. Laboratory investigations showed a normal complete blood count. The urine analysis was normal, and the urine culture was negative.

**Fig. 1:**
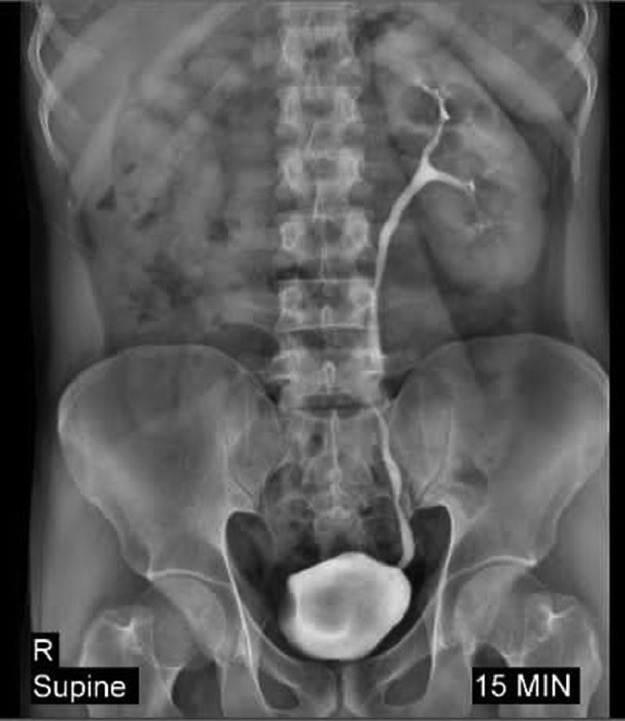
Intravenous pyelogram after 15 minutes showing nonvisualized right kidney and compensatory hypertrophy of the contralateral kidney.

**Fig. 2:**
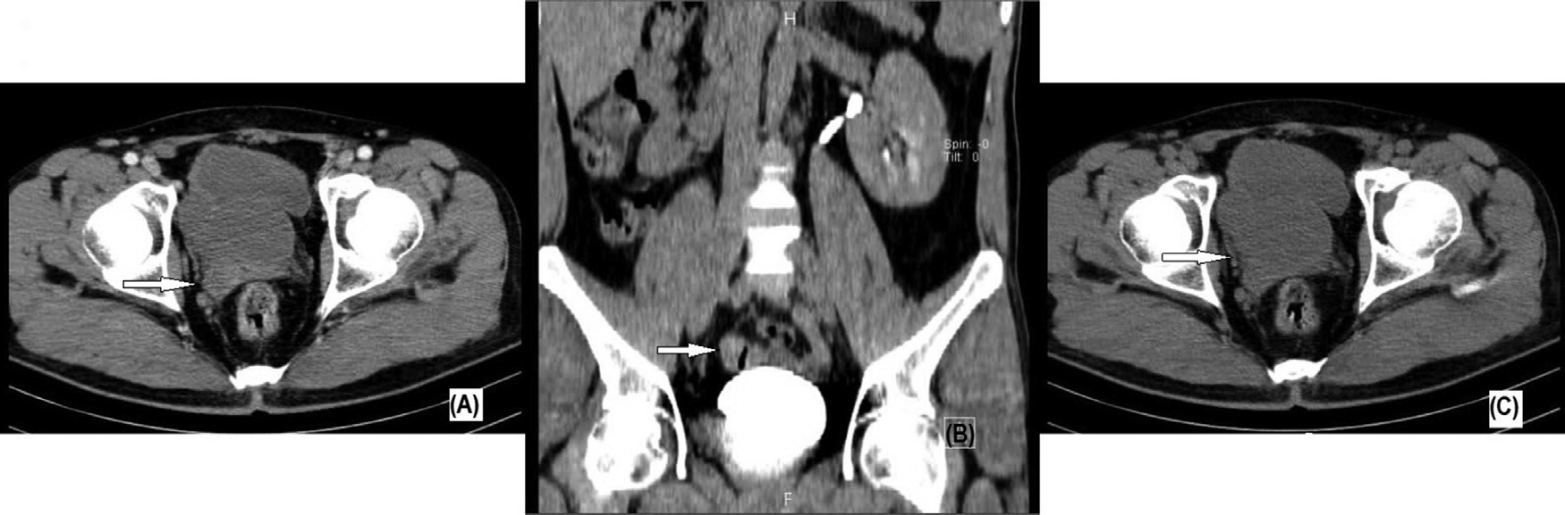
Axial (C), coronal (B), and postcontrast axial (A) CT urography demonstrating right seminal vesicle cystic changes (white arrow).

For further evaluation, an abdominopelvic ultrasound was performed which showed a hypertrophic left kidney measuring 145 × 22 mm due to empty right renal fossa. In addition, a cystic area was identified in the right ureterovesical junction. Intravenous pyelogram (IVP) revealed a nonvisualized right kidney and no contrast enhancement indicative of right renal agenesia and compensatory hypertrophy of the contralateral kidney (Fig. 1). In addition, a suspicious mass effect on the right lateral wall of the bladder in IVP was confirmed by coronal CT urography. CT Scan ± intravenous contrast was performed and determined a large multiloculated multiseptated cystic lesion with a minimal enhancement of septations on the right pelvic side, just posterior to the right side of the bladder, at the place of right seminal vesicle, causing anterior displacement of the bladder, suggestive of right seminal vesicle cystic changes (Figs. 2A, B, C). Zinner syndrome was diagnosed based on the patient's history, radiological findings, and clinical presentation. Analyses of the seminal fluid were reported as azoospermia. conservative treatment was recommenced for pain. As patient was not planning to have children for the moment, we kept him in a follow-up program. Following a 2-week review, after the onset of the initial symptoms, the patient was symptom-free. He was consulted 6 months after the treatment and remained asymptomatic.

## Discussion

Zinner syndrome is caused by an incomplete embryogenesis of the ureteric bud between the fourth and the 13th week of gestation. Females with Mayer-Rokitansky-Küster-Hauser syndrome are considered counterparts of Zinner syndrome [1,6].

The most common symptoms include abdominal pain, fullness, and symptoms related to micturition, such as obstructive urination, dysuria, urgency, and hematuria. Additionally, infertility, painful ejaculation, and recurrent epididymitis have been reported, with most patients presenting between the second and fourth decade of their lives [1]. It is often misinterpreted due to its non-specific signs and lack of symptoms.

Radiology plays a crucial role in the diagnosis and management of Zinner Syndrome. This rare condition is typically diagnosed incidentally during imaging studies, such as ultrasonography and MRI. In cases where Zinner Syndrome is suspected, radiological evaluation is essential for accurate diagnosis and appropriate management [4].

Ultrasonography is often the first imaging modality used to evaluate patients with suspected Zinner Syndrome. This noninvasive technique can detect seminal vesicle cysts and the absence of the ipsilateral kidney, additionally there may be internal echoes in a cyst as a result of infection or hemorrhage. Ultrasonography can also be used to monitor the progression of the cystic lesions and the response to treatment [5,7]. CT scan is another reliable diagnostic option. It is possible to detect renal agenesis and retrovesicular cystic pelvic masses as a result of enlarged seminal vesicles using a CT scan [2,8].

MRI is a more sensitive imaging modality for the diagnosis of Zinner Syndrome. In particular, MRI can provide accurate information about the location, size, and relationship of the cystic lesions to adjacent structures. This information is crucial for surgical planning and management [9]. MRI is useful in identifying seminal vesicle cysts and has a low signal intensity on T1 images and a high signal intensity on T2 images. There can be a high signal intensity on T1 due to hemorrhage or the presence of proteinaceous fluid [10].

Prompt and accurate diagnosis is important to prevent the potential morbidity associated with this condition, such as recurrent epididymitis, urinary tract infections, and infertility [11]. Radiological evaluation can be used to monitor the progression of the cystic lesions and the response to treatment. In some symptomatic cases, surgical intervention may be necessary, and radiology is essential for surgical planning and postoperative monitoring [11,12].

In conclusion, radiology plays a crucial role in the diagnosis and management of Zinner Syndrome. In spite of the fact that ultrasonography can be helpful, CT and MRI are the main diagnostic tools that provide accurate diagnoses of Zinner syndrome.

## Patient consent

A written informed consent for publication of this case was obtained from the patient.
